# Outbreak of influenza A in a boarding school in South Africa, 2016

**DOI:** 10.11604/pamj.2019.33.42.16666

**Published:** 2019-05-21

**Authors:** Jackie Kleynhans, Florette Kathleen Treurnicht, Cheryl Cohen, Theesan Vedan, Mpho Seleka, Lwando Maki, Anne von Gottberg, Kerrigan McCarthy, Wayne Ramkrishna, Meredith McMorrow, Sibongile Walaza

**Affiliations:** 1 Centre for Respiratory Diseases and Meningitis (CRDM), National Institute for Communicable Diseases (NICD) of the National Health Laboratory Service (NHLS), Johannesburg, South Africa; 2 South African Field Epidemiology Training Programme (SA-FETP), NICD of the NHLS, Johannesburg, South Africa; 3 School of Public Health, Faculty of Health Sciences, University of the Witwatersrand, Johannesburg, South Africa; 4 Division of Public Health, Surveillance and Response (DPHSR), NICD of the NHLS, Johannesburg, South Africa; 5 School of Pathology, Faculty of Health Sciences, University of the Witwatersrand, Johannesburg, South Africa; 6 South African National Department of Health (NDoH), Pretoria, South Africa; 7 Influenza Division, Centers for Disease Control and Prevention, Atlanta, Georgia, United States of America; 8 U.S. Centers for Disease Control and Prevention, Pretoria, South Africa

**Keywords:** Boarding school, cohort study, cross-sectional study, influenza, outbreak

## Abstract

**Introduction:**

We investigated an outbreak of influenza-like illness (ILI) at a boarding school in Eastern Cape Province, South Africa. We aimed to confirm the etiological agent, estimate attack rates and identify risk factors for illness.

**Methods:**

We conducted a retrospective cohort study including senior school boarders (n=308). Students with ILI (cough and fever) were identified through school medical records. We also conducted a questionnaire-based cross-sectional study among senior students including boarders (n=107) and day students (n=45). We collected respiratory specimens for respiratory pathogen testing by real-time polymerase chain reaction from a subset of symptomatic students. We calculated attack rates of medically attended ILI (medILI) and identified factors associated with medILI using logistic regression. We calculated seasonal influenza vaccine effectiveness (VE) against medILI.

**Results:**

Influenza A (H3N2) virus was detected in 61% (23/38) of specimens. Attack rate for medILI was 13% among boarders (39/308) in the cohort study and 20% in both day students (9/45) and boarders (21/107) in the cross-sectional study. Playing squash was associated with medILI (aOR 5.35, 95% confidence interval [95% CI]: 1.68-17.07). Of the boarders, 19% (57/308) were vaccinated before the outbreak. The adjusted VE against medILI was 18% (aOR 0.82, 95% CI 0.38-1.78). The outbreak led to cancellation of several events and the need for academic remedial sessions.

**Conclusion:**

We confirmed an influenza A (H3N2) virus outbreak with a high attack rate. The outbreak affected academic and sports activities. Participation in sports and social gatherings while experiencing ILI should be discouraged to reduce viral transmission and impact on school activities.

## Introduction

Influenza-like illness (ILI) and influenza outbreaks commonly occur in schools [1,2], with areas like United Kingdom reporting as many as 658 school respiratory illness outbreaks in one season, with attack rates ranging from 14-45% [3]. There are few data describing outbreaks of influenza or ILI in South Africa. To our knowledge, only three outbreaks have been described in peer-reviewed journals. This includes an outbreak of influenza A (H3N2) virus in a police residential college in Pretoria in 2003 with an attack rate of 20-47% [4], an outbreak affecting students from several schools in Bophuthatswana in 1978 where the strain was not characterized [5], and an outbreak of influenza A (H2N2) virus in gold miners near Johannesburg in 1958 with an attack rate of 15% [6].

On the 14^th^ of July 2016, the resident doctor at a boarding school in the Eastern Cape Province, South Africa, contacted the National Institute for Communicable Diseases (NICD) of the National Health Laboratory Service (NHLS) and reported a large number of students presenting with ILI since the 9^th^ of July, more cases than he had experienced for any influenza season in his time working at the school. Symptoms among students presenting to the sanatorium included fever, cough and generalised body pains. Due to the high number of cases, the disruption of school activities and the unique opportunity to describe an influenza outbreak in a closed population, we conducted an investigation during the 26-29^th^ of July 2016, aimed to confirm the etiological agent, estimate attack rates among students and identify risk factors for illness.

## Methods

The investigation took place at a co-educational private school in the Eastern Cape province. Besides South Africans, the nationalities of the students varied and included more than 20 countries.

Interviews were held with the school sanatorium doctor and nurse, the senior school headmaster, head of academics and head of sport to gather information on the setting and the impact of the outbreak on the school. The outbreak was confined mainly to senior school students and therefore the focus of this investigation was on the senior school students (grades 8-12 and bridge year students). Cases were identified from medical record review and questionnaires administered to staff and students.

### Cohort study

We conducted a cohort study to estimate the attack rate of medically attended ILI (medILI) treated in the school sanatorium. We included all 308 senior school boarding students, but excluded day students as they were more likely to seek private healthcare and we did not have the resources or ethical permission to review records from students at all private practitioners in the town. Cases of medILI from 3 July - 1 August 2016 (start of the school term until end of investigation) were defined as boarding students having a cough and fever as noted on records or extrapolated from prescribed medication, and were identified through a retrospective review of medical records and the admission log at the sanatorium. Fever was defined as either a documented body temperature above 38°C or “fever” noted under symptoms in the medical record. Non-cases were persons who did not meet the medILI case definition. Medical records of 198 patients were reviewed from the senior school boarding population, including all cases of clinician-diagnosed influenza from 3-27 July 2016 (n=82), and a convenience sample of a subset of non-cases (n=116). Age, residence information and influenza vaccination status prior to the outbreak for all boarders were obtained from the school's register. Attack rates were calculated by dividing the number of cases of medILI by the specified population. A multivariable logistic regression analysis was conducted to compare the rate of medILI in each subgroup, including all variables that produced a p-value <0.2 in the univariate analysis. Variables considered in the model were age, gender and boarding house. The vaccine effectiveness against medILI in senior boarders was calculated by determining the adjusted odds ratio (aOR) of medILI in those vaccinated to those not vaccinated and then using the following formula: VE = (1-aOR) x 100.

### Cross-sectional study

We also performed a cross-sectional study to assess the extent of the outbreak and risk factors for illness in a broader population including the day students of the senior school. All senior students (boarding and day) were invited to complete a self-administered, web-based questionnaire which collected information on demographics, travel and medical history, current influenza vaccination status, ILI symptoms and participation in academic, sport and cultural activities in the month prior to the investigation. An epidemiological curve was constructed to include the time period before the start of the term to indicate the baseline level of medILI before the outbreak and the medILI that occurred until the end of the outbreak investigation (25 June - 27 July 2016). Attack rates for self-reported medILI (self-reported cough and fever and seeking medical care from a nurse or clinician) were calculated from the start of the school term (3 July 2016) to when the investigation was performed (27 July 2016) by dividing the number of individuals reporting each condition by the specified population. A multivariable logistic regression analysis compared the proportion reporting medILI in each of the subgroups, assessing all variables with a p-value <0.2 in the univariate analysis. Variables with a p-value <0.05 were included in the final analysis. Variables considered in the model were age, grade, gender, nationality, boarding/day student, boarding house, having cubicles in the boarding room, participation in sports and other activities and having underling medical conditions.

### Data processing and analysis

The web-based questionnaire used in the cross-sectional study was done on the Google forms platform and the output exported to Microsoft Excel 2010 (Microsoft Corporation, Redmond, WA, USA). Data from medical records and the student register collected for the cohort study were captured on the same database. Data were analysed in Stata version 14.0 (StataCorp LLC, College Station, TX, USA).

### Laboratory investigations

Respiratory specimens were collected by the resident doctor and nurse from a convenient subset of boarding and day senior students clinically diagnosed with influenza from 13 to 29 July 2016. The swabs were transported in universal transport medium to the Centre for Respiratory Diseases and Meningitis, NICD, Johannesburg.

Specimens were tested for virological and bacteriological respiratory pathogens. Isolation of nucleic acid was performed by using MagNA Pure 96 System (Roche Diagnostics, Mannheim, Germany) according to the manufacturer's protocol. The first four specimens collected were tested using a real-time reverse-transcription polymerase chain reaction (rtRT-PCR) FTD Respiratory pathogens 21 assay (Fast Track Diagnostics Ltd., Sliema, Malta) for the detection of influenza A viruses, influenza B viruses, rhinoviruses, coronaviruses NL63, 229E, OC43, HKU1, parainfluenza viruses 1,2,3,4, human metapneumovirus A/B, bocavirus, respiratory syncytial virus (RSV) A/B, adenoviruses, enteroviruses, parechoviruses and *Mycoplasma pneumonia*. All subsequent specimens were tested using an rtRT-PCR FTD FLU/HRSV assay (Fast Track Diagnostics Ltd.) to test for influenza type A and B viruses and RSV. Influenza A positive specimens were further subtyped by rtRT-PCR using Centers for Disease Control and Prevention (CDC, Atlanta, Georgia, USA) primers and probes specific for H1N1pdm09 and H3N2 viruses following a previously described protocol [7].

Specimens were also tested for eleven bacterial pathogens (*Streptococcus pneumoniae, Staphylococcus aureus, Haemophilus influenzae, Moraxella catarrhalis, Klebsiella pneumoniae, Pseudomonas aeruginosa, Mycoplasma pneumoniae, Chlamydia pneumoniae, Legionella spp, Bordetella pertussis and Neisseria meningitides*) using commercial FTD kits (community-acquired pneumonia [CAP1] and hospital-acquired pneumonia [HAP]; [Fast Track Diagnostics Ltd.) and in-house real-time PCR kits and assays [8-11].

A subset of the laboratory-confirmed influenza A (H3N2) virus specimens (n=19) were sequenced using the Illumina MiSeq platform (Illumina, CA, USA) following enrichment by influenza A virus-specific genome PCR. Genome sequences were assembled using the de novo assembly approach. An amino acid sequence alignment of the complete hemagglutinin (HA) genes excluding the signal peptide for 14 specimens was generated using ClustalW algorithm embedded in BioEdit v7.0.9.1 [12]. The multiple sequence alignment of HA genes (1653 base pairs) of the 2016 Southern Hemisphere recommended A (H3N2) vaccine strain (A/Hong Kong/4801/2014) was included as a reference strain and root, together with 42 South African strains from specimens collected in the period 19 May to 8 August 2016 (retrieved from the Global Initiative on Sharing All Influenza Data [GISAID] database; Annex 1) [13]. The South African specimens were obtained through the NICD influenza surveillance programs and shared with the World Health Organization Collaborating Centres for Reference and Research on Influenza at Francis Crick Institute, London, United Kingdom; Victorian Infectious Diseases Reference Laboratory in Melbourne, Australia and the Centers for Disease Control and Prevention (CDC) in Atlanta, Georgia, USA. A phylogenetic tree was generated by the maximum likelihood method employing the Hasegawa-Kishino-Yano (HKY) evolutionary model in the MEGA (Molecular Evolutionary Genetics Analysis) [14] version 6 program. To determine confidence for support of the branches, 500 bootstrap replicates were employed.

### Ethical considerations

Prior to the investigation, guidance on managing parental consent for this outbreak investigation was sought from the chairperson of the Human Research Ethics Committee (Medical) of the University of the Witwatersrand. The investigation was covered by the clearance certificate for Essential Communicable Diseases Surveillance and Outbreak response investigation activities of the NICD from the Human Research Ethics Committee (Medical) of the University of the Witwatersrand, reference M160667. Permission was obtained from the Eastern Cape Department of Health (EC-2017RP52_694). Informed consent and assent were obtained from parents and students to complete the questionnaires in the cross-sectional study. All information collected was linked to an anonymised study identification number. The raw data were only accessed by the researcher doing the analysis, which was done on a password-protected computer.

## Results

### Setting

The school employed a nurse and offered primary health care services for boarders and day students. A school doctor consulted when required. The school annually offered the Southern Hemisphere influenza vaccine to consenting students as soon as the vaccine was available in South Africa, which was billed to the student's medical aid.

The senior school hosted grade 8 to 12 as well as bridge year students. The bridging year offered students that finished grade 12 an additional year of school before starting tertiary education. There were 427 senior students of which 308 (72%) were boarders, aged 12-19 years. The senior school had four hostels for boys situated on the main campus, housing between 36-45 boys each. There were two hostels for girls each accommodating 59 and 76 students, situated within walking distance from the main campus. There was also a house allocated to female day students, while the male day students were divided amongst the male boarding houses. Day students spent time in these houses between classes and sports activities. The bridge year students had a separate hostel.

Initially, students presenting to the school sanatorium with ILI were admitted to the sick bay which could host 18 individuals, to allow for isolation and to prevent further spread of the virus. When the sanatorium reached capacity, symptomatic students were encouraged to stay within their rooms in the boarding house and to practise good hand hygiene. Alcohol-based hand sanitizer was made available throughout the boarding houses with signs to encourage usage.

It was recommended that symptomatic students not participate in any sporting events, and from 11-24 July there were multiple students that did not attend sports practice due to illness. Several sports events and practices were cancelled. The sports program recommenced on 25 July. Except for sports practices and matches, many other meetings and cultural events had to be rescheduled or cancelled. Although academic classes were not cancelled during the outbreak, remedial days had to be held after the outbreak for students to catch up on their work.

### Cohort study

**Figure 1:**
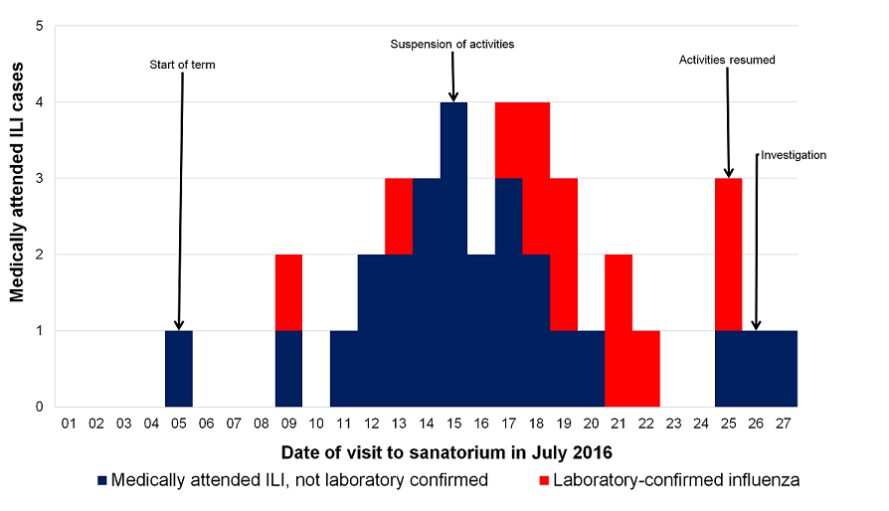
Epidemic curve of medically attended influenza-like illness (ILI, n=39) in senior school boarders in cohort study

We identified 39 medILI cases occurring between 5-27 July (Figure 1). The attack rate of medILI (Table 1) amongst senior boarders was 13% (39/308). There were no significant differences in attack rates by age group, gender or residence. Of 308 boarders, 19% (n=57) were vaccinated prior to the outbreak. Additional vaccinations were not provided during the outbreak. After adjusting for gender, the vaccine effectiveness against medILI was 18% (aOR 0.82, 95%CI 0.38-1.78). Only two cases resulted in complications. There was one hospitalization reported when a student fainted at an event and one case of suspected pneumonia; both students recovered.

**Table 1:** Description of boarders, medically attended influenza-like illness cases and analysis of potential risk factors for medILI

		Attack rate n/N (%)	Univariate analysis		
			Odds ratio+	95% confidence interval	p-value
**Gender**	Males	18/171(10.5)	1	-	-
	Females	21/137(15.3)	1.24	0.78-3.02	0.21
**Age**	≤15	13/123(10.5)	1	-	-
	>15	26/185(14.1)	1.41	0.69-2.85	0.36
**Residence**	Male residence 1	3/41(7.3)	1	-	-
	Male residence 2	4/42(9.5)	1.30	0.27-6.20	0.74
	Male residence 3	8/45(17.8)	2.67	0.66-10.84	0.72
	Male residence 4	3/35(8.6)	1.16	0.22-6.13	0.87
	Female residence 1	13/76(13.2)	1.87	0.48-7.22	0.37
	Female residence 2	11/58(19.0)	2.89	0.75-11.11	0.12
	Bridge year residence	0/11(0)	0	-	-

+Crude odds ratios displayed. Logistic regression not performed as no factors were significantly associated with medILI

### Cross-sectional study in students

**Figure 2:**
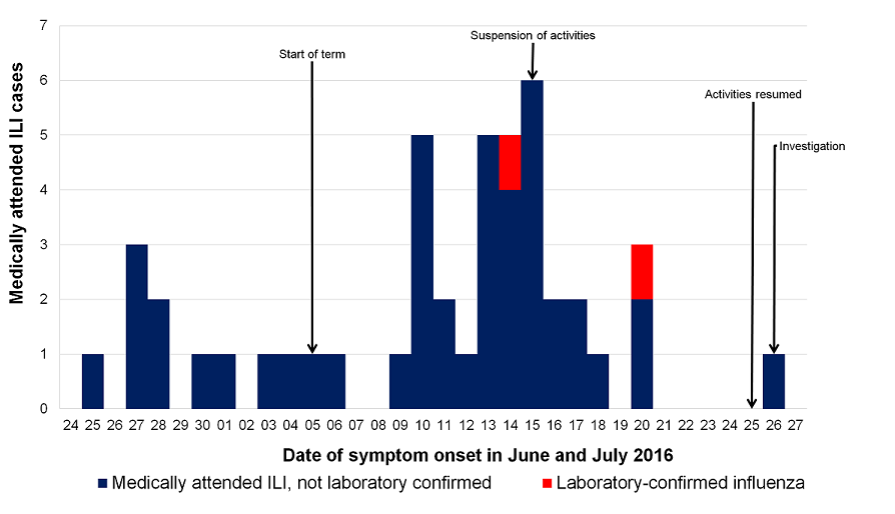
Epidemic curve of self-reported medically attended influenza-like illness (ILI, n=36) in senior school students in cross-sectional study

Of 427 students invited to complete the questionnaire, 36% (n=152) participated. Responses included 35% (107/308) of the boarders and 38% (45/119) of day students. Thirty-six cases were identified from 25 June - 27 July 2016, 30 cases within the outbreak period, attack rate 20% (Figure 2).

On multivariable analysis, playing squash (aOR 5.35, 95%CI 1.68-17.07) and being in grade 8 (aOR 6.84 p=0.05) were significantly associated with medILI (Table 2).

**Table 2:** Description of students that participated in cross-sectional study, attack rates of medically attended influenza-like illness(medILI) and potential risk factors for medILI

		Attack rate n/N(%)	Univariate analysis			Multivariable analysis		
			OR	95% CI	p-value	aOR	95% CI	p-value
Age	≤15	9/53(17)	1	-	-	-	-	-
	> 15	21/99(21)	1.27	0.53-3.05	0.586	-	-	-
Grade	8	5/17(29)	5.21	0.88-30.84	0.07	**6.84**	**1.03-45.26**	**0.05**
	9	2/27(7)	1	-	-	1	-	-
	10	4/27(15)	2.17	0.36-13.01	0.40	1.68	0.26-10.82	0.58
	11	10/40(25)	4.17	0.83-20.87	0.08	4.47	0.82-24.46	0.08
	12	8/36(22)	3.57	0.69-18.42	0.13	3.54	0.64-19.70	0.15
	Bridge year	1/4(25)	4.17	0.28-60.93	0.30	5.49	0.31-95.98	0.31
Gender	Female	19/76(25)	1.97	0.86-4.49	0.107	2.34	0.92-5.98	0.07
	Male	11/76(14)	1	-	-	1	-	-
Nationality	South African	23/114(20)	1.04	0.41-2.69	0.924	-	-	-
	Non-SA	7/36(19)	1	-	-	-	-	-
Boarding	Day student	9/45(20)	1.02	0.43-2.45	0.958	-	-	-
	Boarder	21/107(20)	1	-	-	-	-	-
House	Male res 1	0/11(0)	0	-	-	-	-	-
	Male res 2	3/13(23)	1.20	0.24-6.06	0.825	-	-	-
	Male res 3	4/20(20)	1.00	0.22-4.35	1.000	-	-	-
	Male res 4	1/12(8)	0.36	0.04-3.52	0.382	-	-	-
	Female Res1	8/23(35)	2.13	0.28-7.85	0.254	-	-	-
	Female res 2	5/25(20)	1	-	-	-	-	-
	Female day res	6/27(22)	1.14	0.30-4.34	0.845	-	-	-
	BY res	0/3(0)	0	-	-	-	-	-
Cubicles	No	13/54(24)	2.15	0.70-6.66	0.182	-	-	-
	Yes	5/39(13)	1	-	-	-	-	-
Sport^a^	Athletics	2/22(1)	0.36	0.79-1.64	0.186	-	-	-
	No athletics	28/129(22)	1	-	-	-	-	-
	Basketball	3/16(19)	0.71	0.19-2.66	0.615	-	-	-
	No basketball	27/135(20)	1	-	-	-	-	-
	Cricket	3/35(9)	0.31	0.06-1.77	0.163	-	-	-
	No cricket	8/40(20)	1	-	-	-	-	-
	Hockey	18/92(20)	0.95	0.42-2.16	0.907	-	-	-
	No hockey	12/59(20)	1	-	-	-	-	-
	Netball	10/43(23)	0.81	0.25-2.64	0.689	-	-	-
	No netball	9/33(27)	1	-	-	-	-	-
	Rugby	4/42(10)	0.39	0.08-1.74	0.156	-	-	-
	No rugby	7/33(21)	1	-	-	-	-	-
	Squash	8/22(36)	**2.78**	**1.04-7.42**	**0.041**	**5.35**	**1.68-17.07**	**0.005**
	No squash	22/129(17)	1	-	-	1	-	-
	Swimming	1/13(8)	0.31	0.04-2.51	0.274	-	-	-
	No swimming	29/38(21)	1	-	-	-	-	-
	Tennis	9/32(28)	1.83	0.74-4.51	0.191	-	-	-
	No tennis	21/119(18)	1	-	-	-	-	-
	Water polo	11/41(27)	1.76	0.75-4.11	0.194	-	-	-
	No water polo	19/110(17)	1	-	-	-	-	-
Other Activities^a^	Cheering	11/53(21)	1.09	0.47-2.50	0.841	-	-	-
	No cheering	19/98(19)	1	-	-	-	-	-
	Drama	4/21(20)	0.94	0.29-3.04	0.919	-	-	-
	No drama	26/130(20)	1	-	-	-	-	-
	Music	9/27(30)	2.45	0.97-6.20	0.058	-	-	-
	No music	21/124(17)	1	-	-	-	-	-
Medical History	Underlying illness^b^	7/35(20)	1	-	-	-	-	-
	No under-lying illness^b^	23/117(20)	0.98	0.38-2.52	0.964	-	-	-
Asthma	Asthma	5/31(16)	1	-	-	-	-	-
	No asthma	25/120(21)	1.37	0.47-3.92	0.560	-	-	-

**OR** – Odds ratio, **CI** – confidence interval. Final logistic regression model included gender, grade and squash participation. Reference group underlined.

a For most factors, characteristics were mutually exclusive and associations were calculated in comparison with group underlined. For sport and other activities, associations were calculated individually for each sport/activity(participating vs. not participating) as students participate in multiple sports/activities and groups are therefore not mutually exclusive.

b Underlying illness includes asthma, epilepsy and diabetes.

Of 152 students who completed the questionnaire, 116 days of school were missed due to absenteeism caused by ILI. Among students, 33% (n=50) missed school, 29% (n=44) missed study time, 45% (n=68) missed sport and 14% (n=21) missed other events or time with their friends. The mean time missed from school was 3.4 days per ill student (range 1-11 days). Thirty percent (n=45) of students that completed the questionnaire reportedly received the 2016 vaccine before the outbreak. The reasons provided for not receiving the vaccine are shown in Table 3.

**Table 3:** Reasons cited for not getting vaccinated for influenza

	n	%
People still fall ill after they receive the vaccine	40	26.3
I do not like needles	27	17.8
My parents said I didn’t need to	26	17.1
It will make me sick	20	13.2
I am not at risk for severe influenza	14	9.2
It will be painful	6	4
It is expensive	3	2
Vaccinations are unsafe	2	1.3
I did not get the opportunity	0	0

### Laboratory investigations

**Figure 3:**
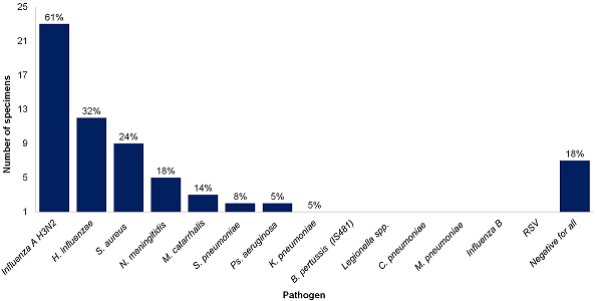
Real-time PCR results for bacterial and viral pathogens tested for in the respiratory specimens collected (n=38)

Of the first four nasal specimens collected before the outbreak investigation, influenza A viruses were detected in all, while all tests for all other pathogens in a 21-plex respiratory pathogens assay were negative. A further 34 respiratory specimens collected (27 nasopharyngeal and 7 throat swabs) from day and boarding students were tested as part of the investigation. Of all the specimens collected (n=38), 23 (61%) tested positive for influenza A (H3N2) viruses. No influenza B viruses or RSV were detected. Results from bacteriological investigations are shown in Figure 3. Due to sample volume constraints, only 33 specimens were tested for *N. meningitides*. Thirteen of the 38 specimens (34%) were collected from individuals that did not meet the medILI case definition, 9 of which tested positive for influenza A (H3N2) virus.

**Figure 4:**
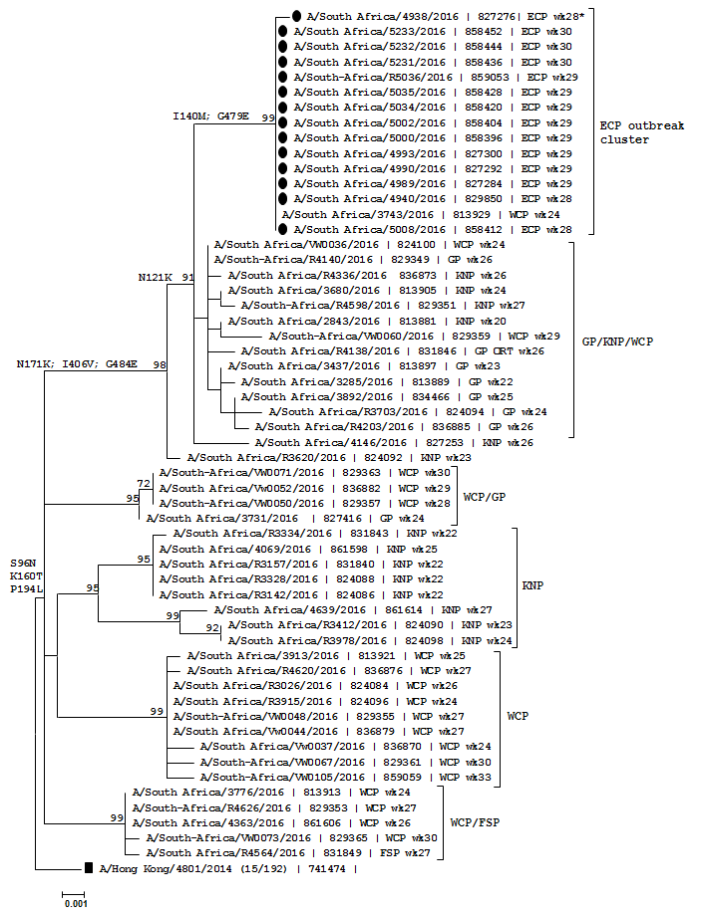
Maximum likelihood phylogenetic tree of the haemagglutinin (HA) gene for 14 of the outbreak specimens (●), 45 South African surveillance and the H3N2 vaccine strain (∎/Hong Kong/4801/2014), South Africa, 2016

On molecular analysis, South African influenza A (H3N2) virus HA gene sequences from the 2016 influenza season formed at least 5 genetic clusters (with geographic clustering pattern) supported by bootstrap values of 98-100% and belong to the 3C.2a genetic clade (Figure 4). All HA genes from influenza A (H3N2) viruses identified in the school outbreak were in the genetic cluster characterised by the following amino acid mutations when compared to the 2016 vaccine strain: N171K, I406V and G484E. The outbreak strains formed a distinct highly homogeneous monophyletic sub-cluster with 99% bootstrap support characterised by N121K, I140M and G479E substitutions when compared to the vaccine strain. Included in the outbreak sub-cluster was a single H3N2 virus originally identified in an individual from the Western Cape Province collected in week 24 of the 2016 season trough routine surveillance. All outbreak specimens were collected in weeks 28 to 30. This Eastern Cape Province lineage was distinguished from the A/Hong Kong/4801/2014 vaccine strain by a total of 9 amino acid mutations including the aforementioned and following mutations in the deduced HA amino acid sequences (excluding signal peptide): S96N, K160T and P194L. These 9 mutations are characteristic of the recently emerged 3C.2a1 sub-lineage. All other South African viruses used as reference belong, like the vaccine strain, to clade 3C.2a.

## Discussion

The outbreak occurred from 9-28 July based on the epidemiological curve and the last samples that tested positive for influenza A (H3N2) virus. Laboratory testing identified influenza A (H3N2) virus as the etiological agent with an overall high attack rate of medILI (13% in cohort and 20% in cross-sectional study), with participating in the sport squash being the strongest risk factor associated with medILI. Multiple sporting and cultural events were cancelled during the outbreak and academic remedial sessions were rescheduled afterwards.

There are several non-influenza viruses that can cause ILI, as well as some bacterial pathogens, although bacterial aetiologies for ILI are rare [15]. The most common pathogens isolated in non-influenza virus ILI include rhinoviruses, parainfluenza viruses, respiratory syncytial virus, human metapneumovirus, adenoviruses and coronaviruses [16,17], none of which were identified during laboratory testing of the specimens. Although bacterial pathogens were detected in some samples, levels were consistent with colonization of the respiratory tract; therefore, it was difficult to determine causation [18,19].

Closed spaces facilitate the spread of influenza viruses since they are transmitted by droplets and aerosols from infected people when coughing or sneezing [20]. A modelling study by Glass *et al.* among school-aged children predicted that social interactions with friends, attending school lectures, and participating in sports were some of the most important factors for influenza transmission [21]. This may be the cause for the association of medILI with playing squash. The sport takes place in a confined space leading to participants being in close proximity. Squash is an aerobic sport with associated increases in the heart and respiratory rate. Studies have shown that strenuous exercise can lead to the suppression of the immune system and make individuals more vulnerable to infections, especially of the upper respiratory tract [22,23]. Studies have also shown that individuals may start shedding the virus and become infective up to two days prior to showing symptoms [24]. Asymptomatic shedding may lead to higher levels of transmission.

There was low influenza vaccine coverage for the 2016 season amongst the boarders (18%). Since senior school students and boarding school residents are not listed under the priority groups for influenza vaccination in South Africa [25] the low vaccination coverage is not unexpected. The school does have an influenza vaccination program that encourages vaccination; however, parents and students decide whether or not to be vaccinated. The main reasons students were not vaccinated was the perception that the vaccine doesn't work, being afraid of needles and because parents did not encourage students to be vaccinated.

The VE against medILI in this study was 18%, in keeping with results from national influenza surveillance. The South African national VE estimates against laboratory-confirmed influenza for the 2016 Southern Hemisphere season for all influenza subtypes was 18% (95%CI minus 55%-60%), and 0.4% (95%CI minus 124%-56%) against influenza A (H3N2) viruses [26]. The influenza strain that circulated during this outbreak may have undergone antigenic drift from the vaccine strain leading to reduced protection, but antigenic data were unavailable. Genome sequencing of the strains did indicate some mutations from the vaccine strain, but these mutations would not necessarily translate to a reduction in vaccine effectiveness. The clonal nature of the viruses characterised together with its distinct geographic clustering strongly support the thought that an H3N2 virus from an initial sentinel case may be the cause of the outbreak in this closed school setting.

Despite the low vaccine effectiveness observed, there is overwhelming evidence from the literature indicating that influenza vaccination effectively prevents influenza. Prior studies have shown that influenza vaccination programs in boarding schools can reduce the attack rate of ILI by 50% [27]. Since complications due to influenza rarely arise in healthy school-going children [28], reasons to encourage vaccination in this population should focus on reducing the disruption to academic and sports programs by reducing absenteeism [29] and the indirect effect on reducing the community acquisition of the virus by preventing the amplification of transmission in schools [30,31]. To prevent transmission of influenza in schools and other settings, it is recommended that individuals with ILI symptoms not attend work or school until at least 24 hours after fever subsides. Students, parents and staff should be educated on the differences between a common cold and influenza to ensure that health care is sought if they experience ILI. Furthermore, one should avoid close contacts; limit visitors; wash hands with soap and water or use an alcohol-based hand rub regularly; and wipe down regularly touched surfaces with a disinfectant. Lastly, it is important to practice good respiratory etiquette by covering the mouth and nose with a tissue when coughing or sneezing [32]. Schools should have an outbreak management plan in place to be able to detect when the number of cases exceeds the normal seasonal number for early outbreak detection and have a contingency plan in place if this occurs. This plan should include procedures on how and where to isolate symptomatic students, how to record all cases and how to effectively communicate to staff, students and their parents to ensure cooperation and prevent anxiety.

This study had limitations. The low number of cases contributed to low precision of the VE estimate. We employed a broad case definition and an outcome of laboratory-confirmed influenza would have been more specific but reduced the number of cases available for analysis as not all cases were laboratory tested. The higher attack rate observed in the cross-sectional study may be due to the use of self-reported symptoms and not physician indicate symptoms as in the cohort study. The impact of the outbreak might not be completely represented when using the medically attended ILI case definition as described by WHO which relies on the presence of a fever and cough [33]. There were several cases of clinically diagnosed influenza identified through medical record review that did not meet our case definition; mostly due to the absence of a cough. A recent transmission study in Hong Kong showed that 41% of individuals who tested positive for influenza did not present with fever, cough or sore throat, which was the WHO case definition for ILI prior to 2014 [24]. We had a low response rate (36%) in the cross-sectional study. This was influenced by lack of interest among some students and by lack of parental consent for others. This may have resulted in participation bias where students that were not affected by ILI might have been less likely to participate, inflating the attack rate of ILI in the cross-sectional study. The attack rate for medILI in the cohort study may therefore be a more accurate representation of the outbreak.

## Conclusion

We confirmed an outbreak of influenza A (H3N2) virus in a boarding school possibly initiated by a sentinel case after the return of students from a half-term break. The outbreak impacted academic, cultural and sports activities at the school. Playing squash was associated with medically attended influenza-like illness. Participation in sports activities and attending social gatherings in confined spaces should be discouraged when showing ILI symptoms to prevent infection. Seasonal influenza vaccination should be encouraged in boarding schools to prevent influenza and reduce academic and sports program disruption.

### What is known about this topic

- Influenza-like illness (ILI) and influenza outbreaks commonly occur in schools and can disrupt educational activities;
- There are few data describing outbreaks of influenza or ILI in South Africa.

### What this study adds

- Outbreaks of influenza in schools may have high attack rates and disrupt educational and extracurricular activities;
- Participation in sport activities while infected with influenza A viruses may lead to further spread;
- The most common reason students were not vaccinated was the perception that the vaccine doesn't work.

## Competing interests

The authors declare no competing interests.

## Appendix

**Annex 1:** Sequences information of multiple sequence alignment of HA genes used in the construction of Figure 4

Segment ID	Segment	Country	Collection date	Isolate name	Originating Lab	Submitting Lab		Authors
EPI859059	HA	South Africa	2016-Aug-08	A/South- Africa/VW0105/2016	Sandringham, NICD	Crick Worldwide Influenza Centre		
EPI859055	HA	South Africa	2016-Jul-20	A/South- Africa/R5063/2016	Sandringham, NICD	Crick Worldwide Influenza Centre		
EPI859053	HA	South Africa	2016-Jul-19	A/South- Africa/R5036/2016	Sandringham, NICD	Crick Worldwide Influenza Centre		
EPI859051	HA	South Africa	2016-Jul-19	A/South- Africa/R5034/2016	Sandringham, NICD	Crick Worldwide Influenza Centre		
EPI829365	HA	South Africa	2016-Jul-21	A/South- Africa/VW0073/2016	Sandringham, NICD	Crick Worldwide Influenza Centre		
EPI829363	HA	South Africa	2016-Jul-21	A/South- Africa/VW0071/2016	Sandringham, NICD	Crick Worldwide Influenza Centre		
EPI829361	HA	South Africa	2016-Jul-20	A/South- Africa/VW0067/2016	Sandringham, NICD	Crick Worldwide Influenza Centre		
EPI829359	HA	South Africa	2016-Jul-16	A/South- Africa/VW0060/2016	Sandringham, NICD	Crick Worldwide Influenza Centre		
EPI829357	HA	South Africa	2016-Jul-04	A/South- Africa/VW0050/2016	Sandringham, NICD	Crick Worldwide Influenza Centre		
EPI829355	HA	South Africa	2016-Jun-30	A/South- Africa/VW0048/2016	Sandringham, NICD	Crick Worldwide Influenza Centre		
EPI829353	HA	South Africa	2016-Jul-06	A/South- Africa/R4626/2016	Sandringham, NICD	Crick Worldwide Influenza Centre		
EPI829351	HA	South Africa	2016-Jul-05	A/South- Africa/R4598/2016	Sandringham, NICD	Crick Worldwide Influenza Centre		
EPI829349	HA	South Africa	2016-Jun-27	A/South- Africa/R4140/2016	Sandringham, NICD	Crick Worldwide Influenza Centre		
EPI824100	HA	South Africa	2016-Jun-04	A/South	Sandringham,	Crick Worldwide		
				Africa/VW0036/2016	NICD	Influenza Centre		
EPI824098	HA	South Africa	2016-Jun-14	A/South Africa/R3978/2016	Sandringham, NICD	Crick Worldwide Influenza Centre		
EPI824096	HA	South Africa	2016-Jun-17	A/South Africa/R3915/2016	Sandringham, NICD	Crick Worldwide Influenza Centre		
EPI824094	HA	South Africa	2016-Jun-13	A/South Africa/R3703/2016	Sandringham, NICD	Crick Worldwide Influenza Centre		
EPI824092	HA	South Africa	2016-Jun-08	A/South Africa/R3620/2016	Sandringham, NICD	Crick Worldwide Influenza Centre		
EPI824090	HA	South Africa	2016-Jun-06	A/South Africa/R3412/2016	Sandringham, NICD	Crick Worldwide Influenza Centre		
EPI824088	HA	South Africa	2016-Jun-02	A/South Africa/R3328/2016	Sandringham, NICD	Crick Worldwide Influenza Centre		
EPI824086	HA	South Africa	2016-May-30	A/South Africa/R3142/2016	Sandringham, NICD	Crick Worldwide Influenza Centre		
EPI824084	HA	South Africa	2016-May-26	A/South Africa/R3026/2016	Sandringham, NICD	Crick Worldwide Influenza Centre		
EPI836885	HA	South Africa	2016-Jun-27	A/South Africa/R4203/2016	Sandringham, NICD	WHO Collaborating Centre for Reference and Research on Influenza		Deng,Y-M.; Iannello,P.; Spirason,N.; Lau,H.; Komadina,N.
EPI836882	HA	South Africa	2016-Jul-06	A/South Africa/Vw0052/2016	Sandringham, NICD	WHO Collaborating Centre for Reference and Research on Influenza		Deng,Y-M.; Iannello,P.; Spirason,N.; Lau,H.; Komadina,N.
EPI836879	HA	South Africa	2016-Jun-21	A/South Africa/Vw0044/2016	Sandringham, NICD	WHO Collaborating Centre for Reference and Research on Influenza		Deng,Y-M.; Iannello,P.; Spirason,N.; Lau,H.; Komadina,N.
EPI836876	HA	South Africa	2016-Jul-06	A/South Africa/R4620/2016	Sandringham, NICD	WHO Collaborating Centre for Reference and Research on Influenza		Deng,Y-M.; Iannello,P.; Spirason,N.; Lau,H.; Komadina,N.
EPI836873	HA	South Africa	2016-Jun-30	A/South Africa/R4336/2016	Sandringham, NICD	WHO Collaborating Centre for Reference and Research on		Deng,Y-M.; Iannello,P.; Spirason,N.; Lau,H.;
							Influenza	Komadina,N.
EPI836870	HA	South Africa	2016-Jun-06	A/South Africa/Vw0037/2016	Sandringham, NICD		WHO Collaborating Centre for Reference and Research on Influenza	Deng,Y-M.; Iannello,P.; Spirason,N.; Lau,H.; Komadina,N.
EPI831849	HA	South Africa	2016-Jul-06	A/South Africa/R4564/2016	Sandringham, NICD		WHO Collaborating Centre for Reference and Research on Influenza	
EPI831846	HA	South Africa	2016-Jun-27	A/South Africa/R4138/2016	Sandringham, NICD		WHO Collaborating Centre for Reference and Research on Influenza	
EPI831843	HA	South Africa	2016-Jun-02	A/South Africa/R3334/2016	Sandringham, NICD		WHO Collaborating Centre for Reference and Research on Influenza	
EPI831840	HA	South Africa	2016-May- 30	A/South Africa/R3157/2016	Sandringham, NICD		WHO Collaborating Centre for Reference and Research on Influenza	
EPI861614	HA	South Africa	2016-Jul-06	A/South Africa/4639/2016	Sandringham, NICD		Centers for Disease Control and Prevention	
EPI861606	HA	South Africa	2016-Jun-29	A/South Africa/4363/2016	Sandringham, NICD		Centers for Disease Control and Prevention	
EPI861598	HA	South Africa	2016-Jun-23	A/South Africa/4069/2016	Sandringham, NICD		Centers for Disease Control and Prevention	
EPI858452	HA	South Africa	2016-Jul-25	A/South Africa/5233/2016	Sandringham, NICD		Centers for Disease Control and Prevention	
EPI858444	HA	South Africa	2016-Jul-25	A/South Africa/5232/2016	Sandringham, NICD		Centers for Disease Control and Prevention	
EPI858436	HA	South Africa	2016-Jul-25	A/South Africa/5231/2016	Sandringham, NICD		Centers for Disease Control and Prevention	
EPI858428	HA	South Africa	2016-Jul-19	A/South Africa/5035/2016	Sandringham, NICD		Centers for Disease Control and Prevention	
EPI858420	HA	South Africa	2016-Jul-19	A/South	Sandringham,		Centers for Disease	
				Africa/5034/2016	NICD		Control and Prevention	
EPI858412	HA	South Africa	2016-Jul-16	A/South Africa/5008/2016	Sandringham, NICD		Centers for Disease Control and Prevention	
EPI858404	HA	South Africa	2016-Jul-18	A/South Africa/5002/2016	Sandringham, NICD		Centers for Disease Control and Prevention	
EPI858396	HA	South Africa	2016-Jul-18	A/South Africa/5000/2016	Sandringham, NICD		Centers for Disease Control and Prevention	
EPI834466	HA	South Africa	2016-Jun-30	A/South Africa/3892/2016	Sandringham, NICD		Centers for Disease Control and Prevention	
EPI829850	HA	South Africa	2016-Jul-13	A/South Africa/4940/2016	Sandringham, NICD		Centers for Disease Control and Prevention	
EPI827416	HA	South Africa	2016-Jun-14	A/South Africa/3731/2016	Sandringham, NICD		Centers for Disease Control and Prevention	
EPI827362	HA	South Africa	2016-Jun-29	A/South Africa/4363/2016	Sandringham, NICD		Centers for Disease Control and Prevention	
EPI827300	HA	South Africa	2016-Jul-18	A/South Africa/4993/2016	Sandringham, NICD		Centers for Disease Control and Prevention	
EPI827292	HA	South Africa	2016-Jul-18	A/South Africa/4990/2016	Sandringham, NICD		Centers for Disease Control and Prevention	
EPI827284	HA	South Africa	2016-Jul-18	A/South Africa/4989/2016	Sandringham, NICD		Centers for Disease Control and Prevention	
EPI827276	HA	South Africa	2016-Jul-13	A/South Africa/4938/2016	Sandringham, NICD		Centers for Disease Control and Prevention	
EPI827269	HA	South Africa	2016-Jul-06	A/South Africa/4639/2016	Sandringham, NICD		Centers for Disease Control and Prevention	
EPI827253	HA	South Africa	2016-Jun-27	A/South Africa/4146/2016	Sandringham, NICD		Centers for Disease Control and Prevention	
EPI827245	HA	South Africa	2016-Jun-23	A/South Africa/4069/2016	Sandringham, NICD		Centers for Disease Control and Prevention	
EPI813929	HA	South Africa	2016-Jun-13	A/South Africa/3743/2016	Sandringham, NICD		Centers for Disease Control and Prevention	
EPI813921	HA	South Africa	2016-Jun-20	A/South Africa/3913/2016	Sandringham, NICD		Centers for Disease Control and Prevention	
EPI813913	HA	South Africa	2016-Jun-16	A/South Africa/3776/2016	Sandringham, NICD		Centers for Disease Control and Prevention	
EPI813905	HA	South Africa	2016-Jun-13	A/South	Sandringham,		Centers for Disease	
				Africa/3680/2016	NICD		Control and Prevention	
EPI813897	HA	South Africa	2016-Jun-06	A/South Africa/3437/2016	Sandringham, NICD		Centers for Disease Control and Prevention	
EPI813889	HA	South Africa	2016-Jun-02	A/South Africa/3285/2016	Sandringham, NICD		Centers for Disease Control and Prevention	
EPI813881	HA	South Africa	2016-May-19	A/South Africa/2843/2016	Sandringham, NICD		Centers for Disease Control and Prevention	
EPI741474	HA	Hong Kong (SAR)	2016-Apr-22	A/Hong Kong/4801/2014 (15/192)			National Institute for Biological Standards and Control (NIBSC)	Nicolson, Carolyn
